# Shengmai Formula suppressed over-activated Ras/MAPK pathway in *C. elegans* by opening mitochondrial permeability transition pore via regulating cyclophilin D

**DOI:** 10.1038/srep38934

**Published:** 2016-12-16

**Authors:** Yan Liu, Dejuan Zhi, Menghui Li, Dongling Liu, Xin Wang, Zhengrong Wu, Zhanxin Zhang, Dongqing Fei, Yang Li, Hongmei Zhu, Qingjian Xie, Hui Yang, Hongyu Li

**Affiliations:** 1 Gansu high throughput screening and creation center for health products, School of Pharmacy, Lanzhou University, Donggang West Road No. 199, Lanzhou 730020, P.R. China; 2 Institute of Microbiology, School of Life Sciences, Lanzhou University, Lanzhou 730000, P.R. China; 3 Institute of Biology, Academy of Sciences, Lanzhou 730000, Gansu province, P.R. China.

## Abstract

Since about 30% of all human cancers contain mutationally activated Ras, down regulating the over-activation of Ras/MAPK pathway represents a viable approach for treating cancers. Over-activation of Ras/MAPK pathway is accompanied by accumulation of reactive oxygen species (ROS). One approach for developing anti-cancer drugs is to target ROS production and their accumulation. To test this idea, we have employed *C. elegans* of *let-60 (gf)* mutant, which contain over-activated *let-60* (the homolog of mammalian *ras*) and exhibit tumor-like symptom of multivulva phenotype, to determine whether anti-oxidants can affect their tumor-like phenotype. Specifically we studied the effect of Shengmai formula (SM), a traditional Chinese medicine that has strong anti-oxidant activity, on the physiology of *let-60 (gf)* mutants. Unexpectedly, we found that SM treatment led to the opening of mitochondrial permeability transition pore by regulating cyclophilin D and then triggered oxidative stress and related signaling pathway activation, including p53, JNK, and p38/MAPK pathways. Finally, SM induced mitochondrial pathway of apoptosis and inhibited the tumor-like symptom of the multivulva phenotype of *let-60(gf)* mutants. Our results provide evidences to support that SM act as a pro-oxidant agent and could serve as a potential drug candidate for combating over-activated Ras-related cancer.

Shengmai formula (SM), a traditional Chinese medicine formula, is composed of Radix Ginseng (*Panax ginseng* C.A. Mey) (RG), Radix Ophiopogonis (*Ophiopogon japonicus* (L.f) Ker-Gawl.) (RO), and Fructus Schisandrae (*Schisandra chinensis* (Turcz.) Baill.) (FS)^1^. It has been used for nearly 1,000 years in China, and is currently listed in the Chinese Pharmacopoeia mainly for treating coronary heart disease^1^,^2^,^3^,^4^. Due to its strong anti-oxidant activity, it also serves as one of a few drugs for treating cardiovascular diseases under emergent conditions^5^,^6^,^7^,^8^. Recent studies have revealed that SM attenuates amyloid-β-induced cytotoxicity of PC-12 cells, extends the lifespan of *C. elegans*, recovers cognitive performance and relieves cerebral oxidative damages in BALB/c mice^3^,^9^,^10^. In addition, SM enhances the anti-tumor effect of chemotherapeutic agents in PC-3, LoVo, MCF-7, MCF-7/ADR, and 7404 cell lines^11^. Also, SM inhibits the metastasis of Lewis lung cancer and M-HP melanoma in mice^12^,^13^. Nonetheless, the underlying mechanism for anti-cancer activity of SM remains to be investigated.

Over-activation of Ras is a major factor in the formation of human malignant tumors^14^,^15^,^16^. Previous studies have shown that cancer cells usually contain elevated levels of ROS, and over-activated Ras is known to be closely associated with increased ROS accumulation^17^,^18^. Thus, one approach for developing anti-cancer therapy is to target ROS production and their accumulation^19^. It is well-known that over 90% ROS are produced in mitochondria^20^. Because of this, mitochondria have been suggested to be a viable drug target for treating cancers. However, none of the mitochondrion-targeting drugs have entered clinical application^21^,^22^. It is necessary to discover new anti-cancer drug candidates such alike. In light of the reported strong anti-oxidant activity of SM, it might have an inhibitory effect on ROS accumulation and the over-activated Ras. This speculation prompted us to test these possibilities and to examine whether SM has an anti-cancer activity^2^,^5^,^6^,^7^,^9^.

In *Caenorhabditis elegans, let-60* is a homologous gene to *ras* in mammals, and Ras/MAPK signaling pathway determines the development of worm vulval^23^. The over-activated Ras/MAPK pathway produces an abnormal multivulva (Muv) phenotype, which can be alleviated by anti-cancer drug candidates^24^. *C. elegans* is known as a powerful tool for screening anti-cancer drug candidates from anti-oxidants and pro-oxidants to suppress over-activated Ras/MAPK pathway^25^,^26^. In this study, we used *C. elegans* mutants to investigate whether SM formula can suppress over-activated Ras/MAPK pathway. We also examined whether SM can affect intracellular ROS and target mitochondrial in order to understand the mechanism of the action of SM. Our results provide evidences to support that SM formula can serve as a potential drug candidate for combating over-activated Ras-related cancer.

## Results

### SM suppressed over-activated Ras/MAPK pathway

**Figure 1:**
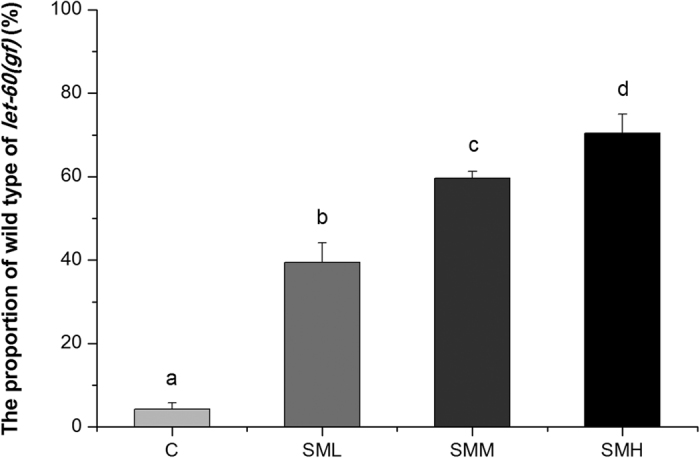
SM significantly inhibited the Muv phenotype of the *let-60 (gf)* mutants in a dose-dependent manner. The Muv phenotype of *let-60 (gf)* mutants were reversed to the wild type phenotype after treated by SM for 3–4 days. The proportion of wild type of *let-60 (gf)* was the percents of wild phenotype worms in tested worms (N = 80–100). C: control. Data is presented as the mean ± SD of at least three independent experiments. Bars with different letters indicated that there was a significant difference at a level of 0.05 among groups.

In *C. elegans, let-60* is the homolog of mammalian *ras, let-60 (gf)* mutation can cause tumor-like symptom of the multivulva phenotype^23^,^24^. Figure 1 showed that SM significantly inhibited the Muv phenotype of *let-60 (gf)* mutants in a dose-dependent manner. *lin-15* and *lin-1* are the upstream and downstream gene of *let-60*, respectively. Both of them are negative regulators of vulval induction, and loss of function of *lin-15* or *lin-1* also causes multivulva phenotype^23^,^27^. According to the genetic epistasis theory, SM should inhibit the Muv phenotype of *lin-15 (lf)* mutants as well. However, SM had no effect on the Muv phenotype of *lin-15 (lf)* (Supplementary Table S1). One possible explanation is that *lin-15* mutants contain a wild type copy of *let-60/ras*, and only over-activated Ras can respond to SM. SM again had no effect on the *lin-1(lf)* mutants, indicating that SM inhibiting Muv phenotype was mechanism based rather than cytotoxicity. Further, SM inhibited Muv phenotype of parent worms with over-activated *ras*, but it did not affect tumor-like symptom of progeny, suggesting that the effect of SM on parent worms was only at the phenotypical level, and not genetic level (Supplementary Fig. S1).

SM is composed of three component herbs of RG, RO and FS. To dissection of SM, each single herb and the possible combinations of them were used to test the inhibitory effect on the Muv phenotype of over-activated Ras mutants. Our results indicated that each herb and all the combinations promoted the wild type proportion of the tested *let-60 (gf)* mutants. In addition, the inhibitory capacity of any combination could be added by the third herb. For example, RG further added the inhibitory activity of RO + FS. It indicated that none of the three herbs can be reduced and they exerted an additive or synergistic effect (Supplementary Fig. S2).

### SM suppressed over-activated Ras *in vivo* was not associated with its anti-oxidant activity *in vitro*

**Figure 2:**
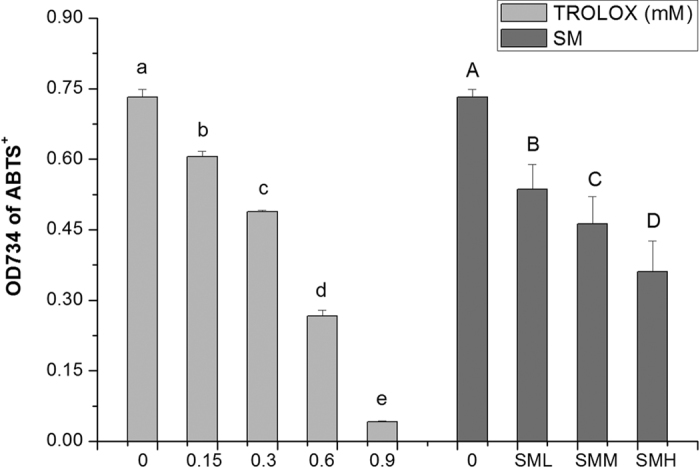
SM had strong anti-oxidant capacity *in vitro*. The anti-oxidant capacity was measured by ABTS method. Data is presented as the mean ± SD of at least three independent experiments. Bars with different letters indicated that there was a significant difference at a level of 0.05 among groups.

**Figure 3:**
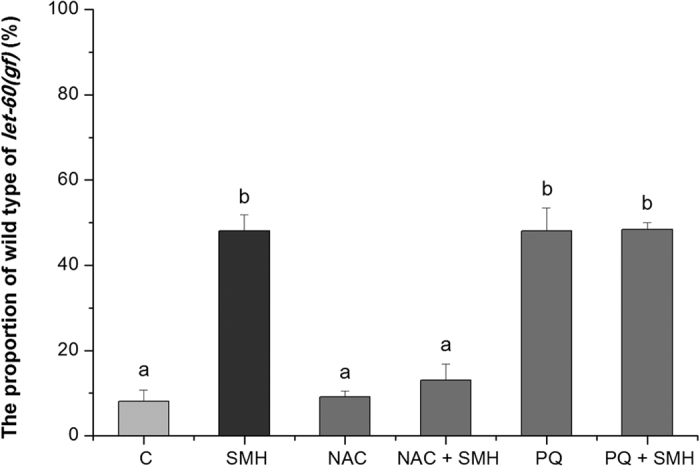
SM inhibiting over-activated Ras could be reverted by NAC, but not PQ. C: control. NAC, 2.5 mM N-Acety-L-Cysteine; PQ, 0.5 mM paraquat. Data is presented as the mean ± SD of at least three independent experiments. Bars with different letters indicated that there was a significant difference at a level of 0.05 among groups.

As expected, Fig. 2 showed that SM significantly reduced ROS *in vitro*. Unexpectedly, N-acety-L-cysteine (NAC), which is an anti-oxidant that can scavenge all kinds of ROS, significantly reversed the inhibitory effects of SM on Muv phenotype of *let-60 (gf)* mutants. Moreover, we found that paraquat (PQ), a well-known pro-oxidant, did not potentiate the inhibitory effect of SM on Muv phenotype of *let-60 (gf)* mutants, suggesting that SM might function in a similar pathway as PQ (Fig. 3). These results would suggest that the anti-oxidant activity of SM shown *in vitro* was not related to its inhibitory effect on over-activation of Ras *in vivo*.

**Figure 4:**
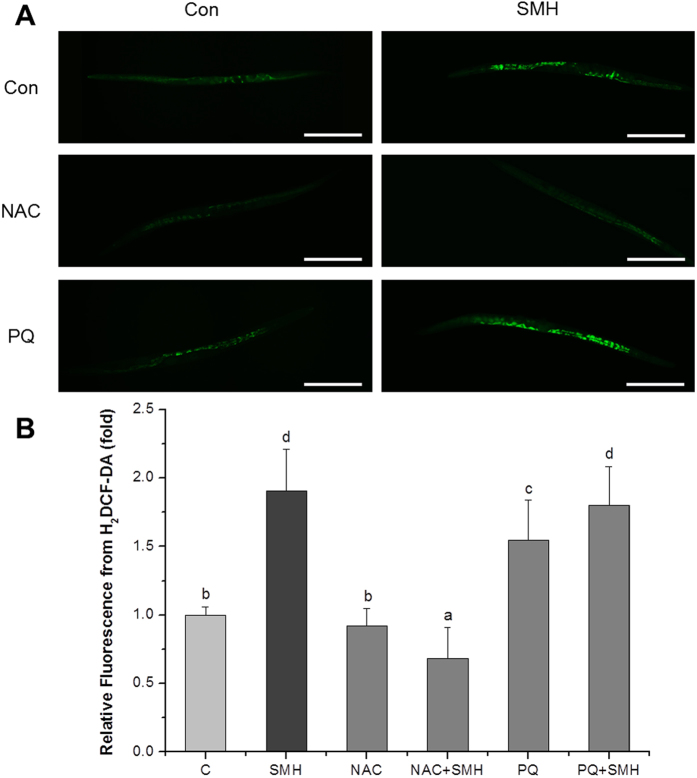
SM increased the level of ROS accumulation in *let-60 (gf)* mutants. (**A**) Fluorescence image of worms, which were stained by 10 μM H_2_DCF-DA at 20 °C for 20 min (N = 20). Con and C: control. NAC, 2.5 mM N-Acety-L-Cysteine; PQ, 0.5 mM paraquat. Scale bar, 200 μm. (**B**) Quantified fluorescence intensity of H_2_DCF-DA of each group. SM significantly increased the ROS accumulation. Data is presented as the mean ± SD of at least three independent experiments. Bars with different letters indicated that there was a significant difference at a level of 0.05 among groups.

To further clarify the role of ROS in the action of SM *in vivo*, we measured the ROS level in *C. elegans* mutants after SM treatment, using the ROS probe H_2_DCF-DA. Figure 4 showed that SM treated animals exhibited an enhanced level of ROS accumulation indeed.

### SM suppressed over-activated Ras via superoxide oxidative stress

**Figure 5:**
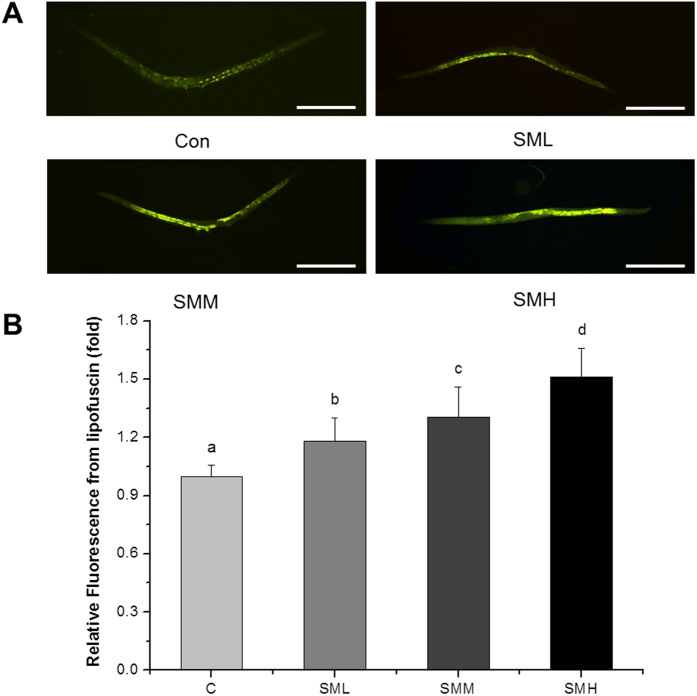
SM increased lipofuscin accumulation in *let-60 (gf)* mutants. (**A**) Fluorescence image of worms after SM treatment for 5 days (N = 20). Con and C: control. Scale bar, 200 μm. (**B**) Quantified fluorescence intensity of lipofuscin. Data is presented as the mean ± SD of at least three independent experiments. Bars with different letters indicated that there was a significant difference at a level of 0.05 among groups.

Next, we employed lipofuscin, an indicator of oxidative stress, to determine whether SM treatment induced oxidative stress in *let-60 (gf)* mutants^28^. As showed in Fig. 5, SM treatment caused a significant increase in the accumulation of lipofuscin in *let-60(gf)* mutants, indicating that SM triggered an oxidative stress. We also measured DAF-16::GFP nuclear translocation (Supplementary Fig. S3), the expression of GST-4 (Supplementary Fig. S4), SOD-1 (Supplementary Fig. S5), SOD-3 (Supplementary Fig. S6), HSP-16.2 (Supplementary Fig. S7), and the SOD activity (Supplementary Fig. S8) and found that all these parameters were up-regulated by SM. These results further supported the notion that SM induced oxidative stress in *C. elegans*.

**Figure 6:**
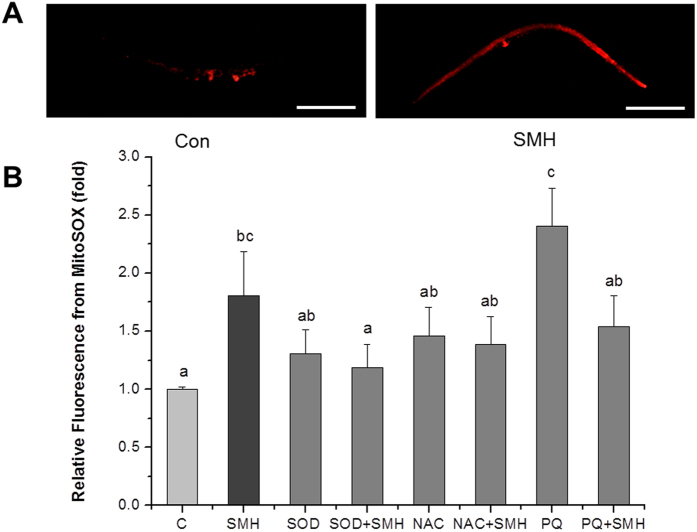
SM significantly increased the level of superoxide in *let-60 (gf)* mutants. (**A**) Fluorescence image of worms after stained by 5 μM MitoSox at 20 °C for 20 min (N = 20). Con and C: control. NAC, 2.5 mM N-Acety-L-Cysteine; PQ, 0.5 mM paraquat. SOD, 150 U superoxide dismutase. Scale bar, 200 μm. (**B**) Quantified fluorescence intensity of MitoSox of each group. Worms treated by SM had a brighter fluorescent level. Data is presented as the mean ± SD of at least three independent experiments. Bars with different letters indicated that there was a significant difference at a level of 0.05 among groups.

According to our knowledge, 90% ROS are produced in mitochondrial, and superoxide is the center of ROS generation. Thus, we further investigated whether SM affected the superoxide level by using MitoSox as a superoxide anion probe^20^,^29^,^30^. Figure 6 showed that SM dramatically triggered an increase in the superoxide level. As a further proof that superoxide may mediate the action of SM, we showed that the effect of the SM inhibiting Muv phenotype was almost completely abolished by the treatment with exogenous superoxide dismutase (SOD) (Supplementary Fig. S9).

### SM opened permeability transition pore (PTP) by regulating cyclophilin D (CypD)

**Figure 7:**
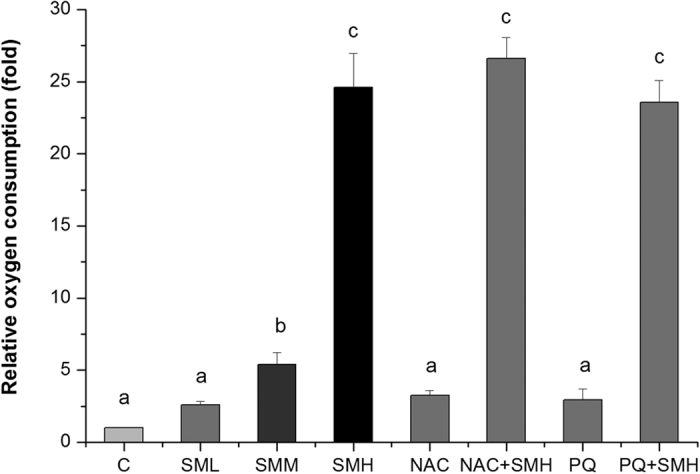
SM increased oxygen consumption in *let-60 (gf)* mutants. Relative oxygen consumption was total oxygen consumption being normalized by protein content measured by the Lowry method after homogenizing worms tested. C: control. NAC, 2.5 mM N-Acety-L-Cysteine; PQ, 0.5 mM paraquat. Data is presented as the mean ± SD of at least three independent experiments. Bars with different letters indicated that there was a significant difference at a level of 0.05 among groups.

**Figure 8:**
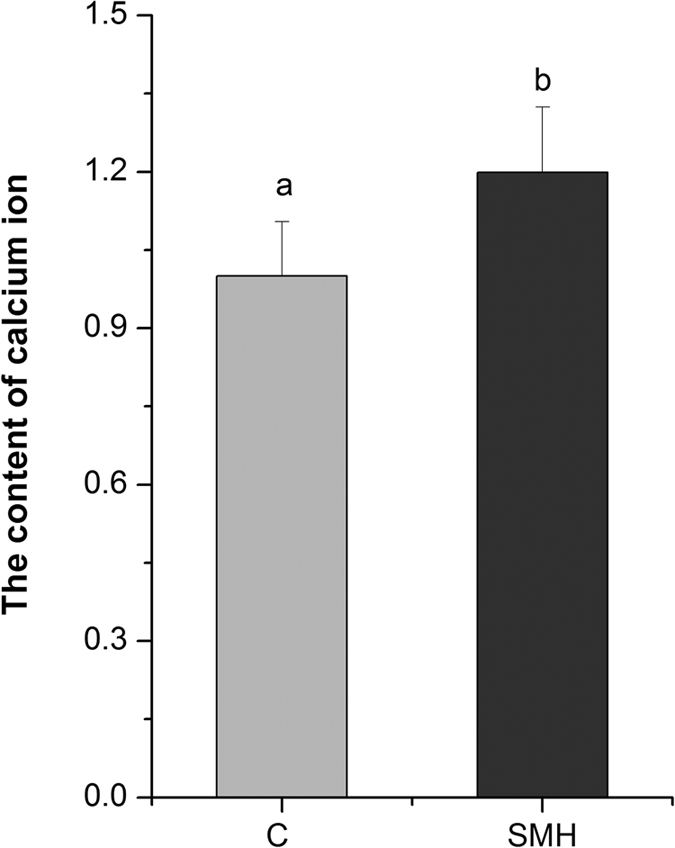
SM increased intracellular free calcium in *let-60 (gf)* mutants. Worms were stained by 5 μM Fluo-3 at 20 °C for 20 min (N = 20). C: control. Data is presented as the mean ± SD of at least three independent experiments. Bars with different letters indicated that there was a significant difference at a level of 0.05 among groups.

**Figure 9:**
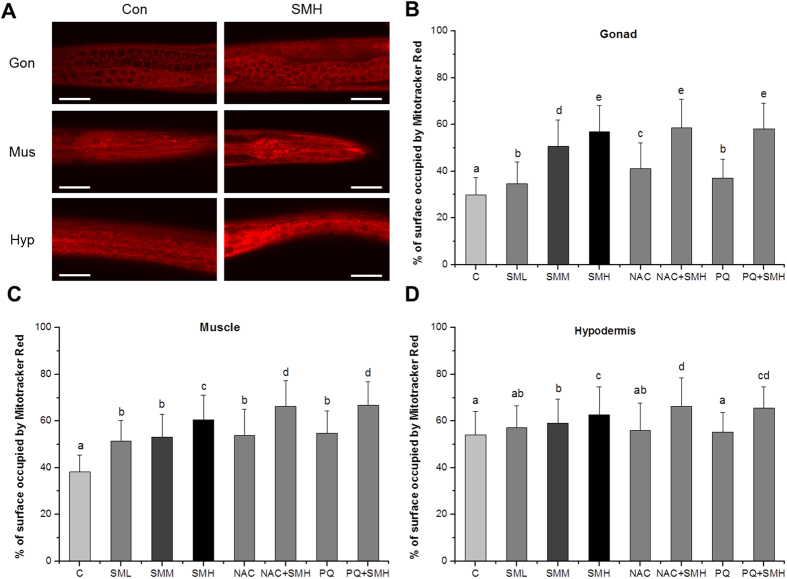
SM increased mitochondrial abundance in *let-60 (gf)* mutants. (**A**) Fluorescence image of different tissues of worms after stained by 5 μM Mitotracker red at 20 °C for 20 min (N = 20). Scale bar, 50 μm. (**B**–**D**) % of the surface occupied by Mitotracker Red fluorescence of the selected region of gonad (Gon), muscle (Mus), hypodermis (Hyp), respectively. Con and C: control. NAC, 2.5 mM N-Acety-L-Cysteine; PQ, 0.5 mM paraquat. Data is presented as the mean ± SD of at least three independent experiments. Bars with different letters indicated that there was a significant difference at a level of 0.05 among groups.

**Figure 10:**
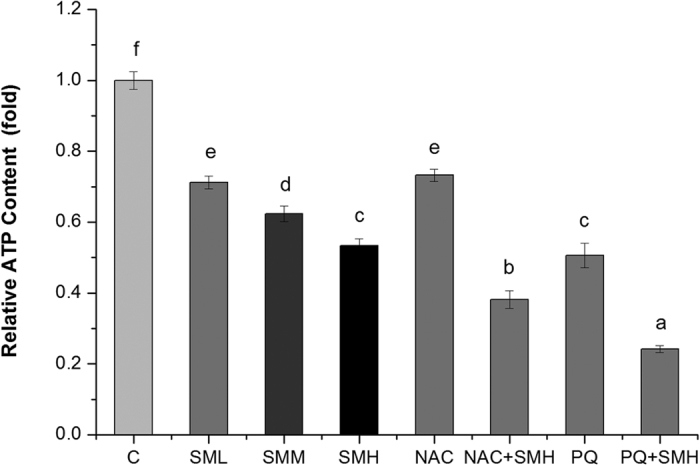
SM significantly inhibited ATP content in *let-60 (gf)* mutants. Relative ATP content was total ATP content being normalized by protein content measured by Lowry method after homogenizing worms tested. C: control. NAC, 2.5 mM N-Acety-L-Cysteine; PQ, 0.5 mM paraquat. Data is presented as the mean ± SD of at least three independent experiments. Bars with different letters indicated that there was a significant difference at a level of 0.05 among groups.

**Figure 11:**
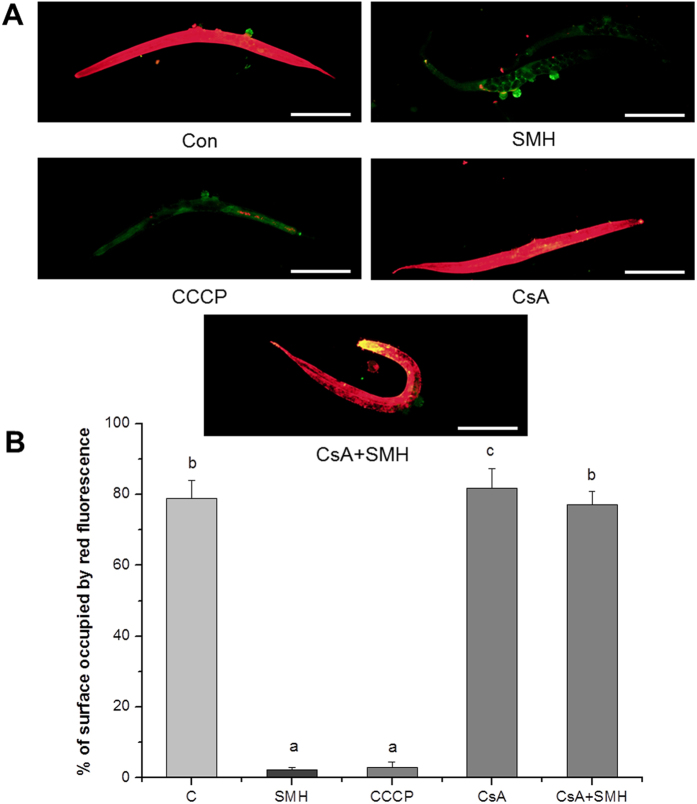
SM significantly decreased mitochondrial membrane potential. (**A**) Fluorescence image of worms were stained by JC-1 at 20 °C for 20 min (N = 20). C: control. CsA, 5 μM Cyclosporin A; CCCP, 10 μM Carbonyl cyanide 3-chlorophenylhydrazone, positive control. Scale bar, 200 μm. (**B**) Quantified fluorescence intensity of JC-1 of each group. Data is presented as the mean ± SD of at least three independent experiments. Bars with different letters indicated that there was a significant difference at a level of 0.05 among groups.

**Figure 12:**
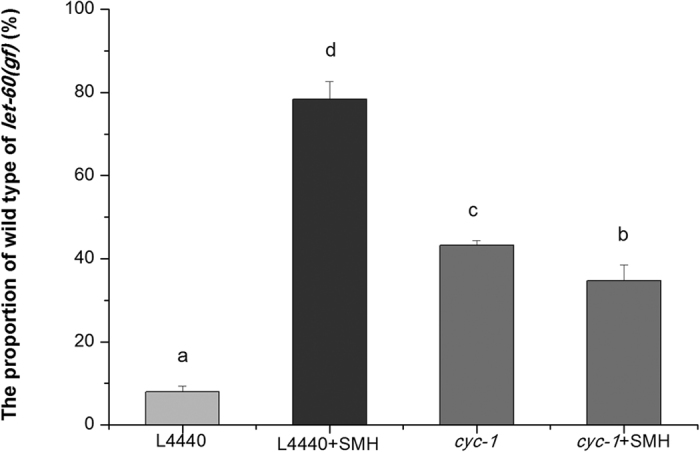
c*yc-1* RNAi reverted the action of SM. 1 mg/mL RNAi bacteria was added to per well and treated the worms for 3–4 days (N = 80–100). HT115 containing empty L4440 was fed as a control. Data is presented as the mean ± SD of at least three independent experiments. Bars with different letters indicated that there was a significant difference at a level of 0.05 among groups.

SM induced superoxide and then elevated ROS in mitochondria. Next, we examined the effect of SM on mitochondrial function. We found that SM increased oxygen consumption (Fig. 7), calcium level (Fig. 8) and mitochondrial abundance (Fig. 9) in *let-60 (gf)* mutants. In contrast, SM decreased ATP level (Fig. 10) and mitochondrial membrane potential (MMP) (Fig. 11). Additionally, we performed RNA interference assay targeting at *cyc-1*, which encodes cytochrome C reductase (complex III) gene, to examine the effect of SM on cytochrome C^31^. As showed in Fig. 12, although *cyc-1* interference increased the proportion of the wild type worms, it blocked the effect of SM on Ras. The result indicated that SM inhibiting *ras* needed intact mitochondrial electronic respiratory chain.

**Figure 13:**
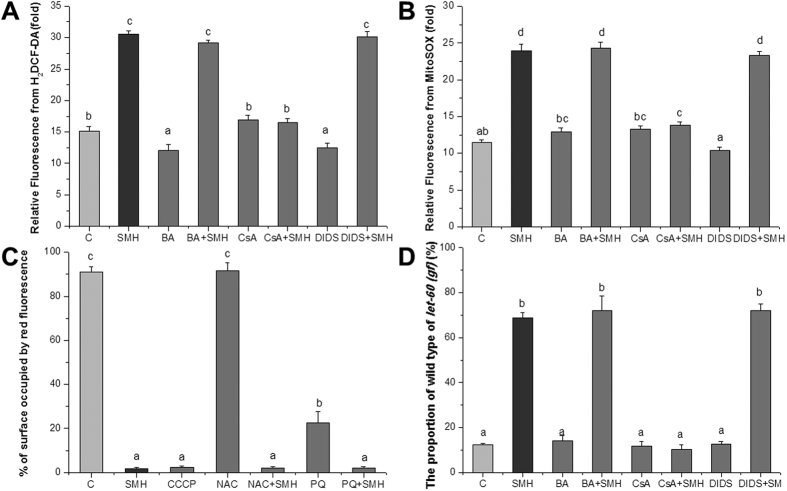
The opening of PTP promoted ROS generation and inhibited the over-activated Ras/MAPK pathway. (**A**) Quantified fluorescence intensity of H_2_DCF-DA (N = 20). (**B**) Quantified fluorescence intensity of MitoSox (N = 20). (**C**) Quantified fluorescence intensity of JC-1 (N = 20). C: control. BA, 8 μM Bongkrekic acid; CsA, 5 μM Cyclosporin A; DIDS, 500 μM 4,4′-Diisothiocyanatostilbene -2,2′-disulfonic acid disodium salt hydrate; CCCP, 10 μM Carbonyl cyanide 3- chlorophenylhydrazone, positive control. (**D**) The effect of PTP inhibitor CsA, BA and DIDS on the Muv phenotype of *let-60 (gf)* mutants (N = 80–100). HT115 containing empty L4440 was fed as a control. CsA completely reversed the effect of SM, but not BA and DIDS. Data is presented as the mean ± SD of at least three independent experiments. Bars with different letters indicated that there was a significant difference at a level of 0.05 among groups.

The accumulation of ROS can trigger PTP opening^32^. By turns, PTP opening can induce ROS accumulation^33^. It is imperative to investigate that ROS accumulation is the cause or the effect of PTP opening. We used ROS probe H_2_DCF-DA and superoxide anion probe MitoSox to examine the ROS and superoxide anion level after closed the three subunits of PTP by their specific inhibitors. Bongkrekic acid (BA), Cyclosporin A (CsA) and 4,4′-Diisothiocyanatostilbene-2,2′-disulfonic acid disodium salt hydrate (DIDS) are subunit inhibitors of the bongkrekic acid-sensitive adenine nucleotide translocase (ANT), CypD and voltage-dependent anion channel (VDAC) of PTP, respectively^34^,^35^. Figure 13A and B showed that, CsA dramatically reversed the accumulation of ROS and superoxide induced by SM in *let-60 (gf)* mutants, but not BA and DIDS. These results indicated that SM regulated the level of ROS and superoxide anion by regulating CypD. Further, we employed MMP probe JC-1 to detect the MMP of *let-60 (gf)* mutants after treated with anti-oxidant NAC and pro-oxidant PQ. Figure 13C showed that NAC did not invert the effect of SM on MMP, indicated that anti-oxidant scavenging ROS did not block PTP opening induced by SM. These results indicated that ROS accumulation was a downstream event of PTP opening. Then, we employed BA, CsA and DIDS to determine the effect of PTP opening induced by SM on the Muv phenotype of the *let-60 (gf)* mutants. Figure 13D showed that, CsA, but not BA and DIDS, completely reversed the anti-Ras over-activation effect of SM.

### Apoptosis and oxidative stress related signaling pathways involved in the action of SM

**Figure 14:**
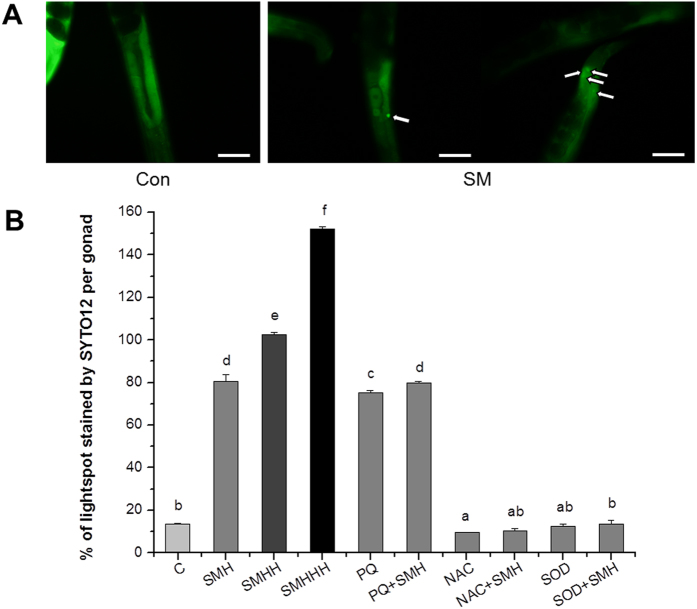
SM enhanced germ cell apoptosis in *let-60 (gf)* mutants. (**A**) Worms were stained by 50 μM SYTO 12 after treated with SM for 48 hours. Germ cell corpse is indicated by white arrows. Con and C: control. SMH, 12 mg/mL; SMHH, 24 mg/mL; SMHHH, 48 mg/mL. NAC, 2.5 mM N-Acety-L-Cysteine; PQ, 0.5 mM paraquat. Scale bar, 50 μm. (**B**) Quantified the number of germ cell corpse of each group. Data is presented as the mean ± SD of at least three independent experiments. Bars with different letters indicated that there was a significant difference at a level of 0.05 among groups.

PTP opening is a hallmark of early apoptosis^36^. In adult *C. elegans*, apoptosis only can be observed in gonad cells^37^. SYTO 12 is a vital dye that preferentially stains apoptotic gonad cells^38^. Therefore, we used SYTO 12 dyes to detect the effect of SM on gonad cell apoptosis. Figure 14 showed SM significantly induced gonad cell apoptosis. Furthermore, the apoptosis induced by SM was completely abolished by treatment with anti-oxidants SOD and NAC. In order to further confirm the effect of SM on gonad cell apoptosis, we also employed AO and Hoechst 33342 double staining to detect the apoptosis cells in gonad. As shown in Supplementary Fig. S10, the results were identified with Fig. 14.

**Figure 15:**
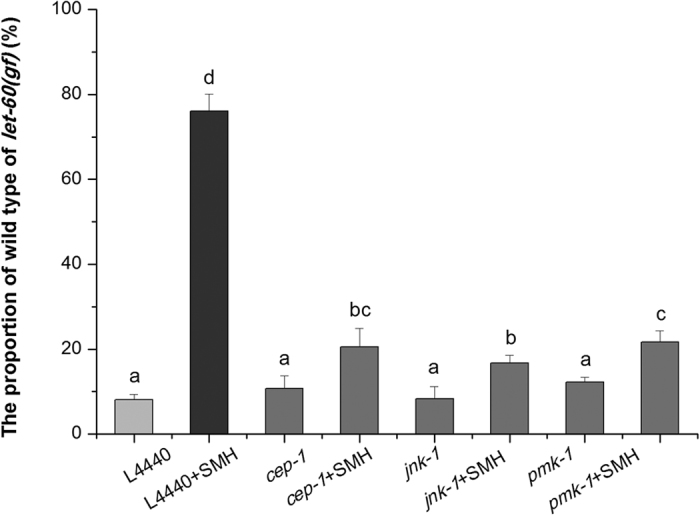
The effect of *cep-1, jnk-1* and *pmk-1* RNAi on the Muv phenotype in *let-60 (gf)* mutants. 1 mg/mL RNAi bacteria was added to per well and treated the worms for 3–4 days (N = 80–100). HT115 containing empty L4440 was fed as a control. p53, JNK, and p38/MAPK pathway are involved in SM down regulating over-activated Ras. Data is presented as the mean ± SD of at least three independent experiments. Bars with different letters indicated that there was a significant difference at a level of 0.05 among groups.

**Figure 16:**
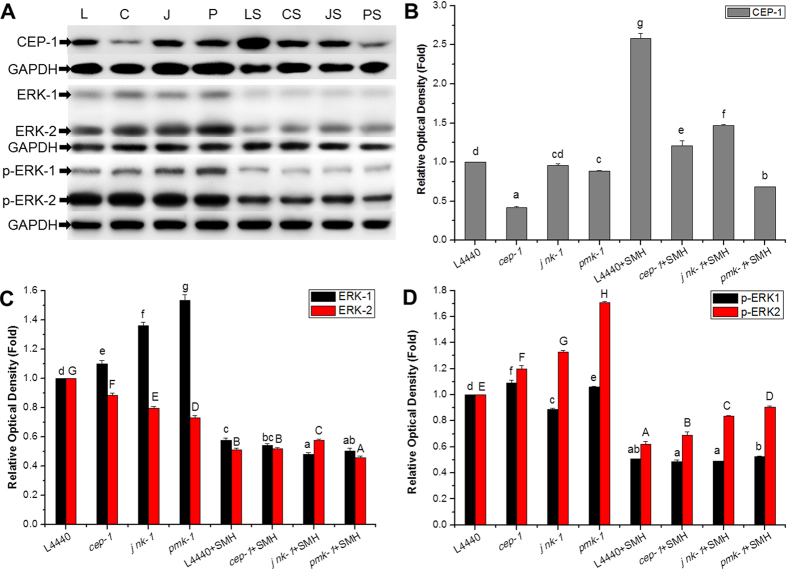
SM inhibited ERK downstream of Ras, and CEP-1 was involved in SM suppressing over-activated Ras/MAPK pathway. (**A**) The effect of SM on ERK1/2, p- ERK1/2 and CEP-1 in *let-60(gf)* mutants. Quantification of CEP-1 (**B**), ERK1/2 (**C**), p-ERK1/2 (**D**) band intensities, which were normalized using GAPDH blots. L, C, J, P, LS, CS, JS and PS were groups treated by RNAi of L4440, *cep-1, jnk-1, pmk-1*, L4440 + SMH, *cep-1* + SMH, *jnk-1* + SMH and *pmk-1* + SMH, respectively. Data is presented as the mean ± SD of at least three independent experiments. Bars with different letters indicated that there was a significant difference at a level of 0.05 among groups.

Since SM treatment induced oxidative stress, related cell stress response signaling pathways should also be activated and involved in SM suppressing over-activated Ras/MAPK pathway, we further tested the effects of RNAi of *cep-1* (homologous to p53), *jnk-1* (homologous to JNK) and *pmk-1* (homologous to p38) on the suppression of Muv phenotype by SM^39^,^40^,^41^,^42^. The results showed that all three gene RNAi partially blocked the inhibitory effect of SM on over-activated *ras*, respectively (Fig. 15). Western blot results showed that SM induced the expression of CEP-1, JNK, p-JNK, p38 and p-p38, and decreased the expression of ERK and p-ERK (Fig. 16 and Supplementary Fig. S11). Our data suggested that SM inhibited the Ras/MAPK pathways, in which p53, JNK, and p38 pathways were all involved.

## Discussion

Intracellular ROS accumulation is one of the major factors for causing and promoting cancer^43^. Suppression of oxidative stress has been considered to be a viable approach in treating cancer^44^. Thus, many anti-oxidant reagents like polyphenols, flavonoids and so on, are considered to be potential anti-cancer drug candidates^45^,^46^,^47^,^48^. Although SM had a strong anti-oxidant activity *in vitro* (Fig. 2), our experiments showed that it enhanced superoxide (Fig. 6) and ROS (Fig. 4) level *in vivo*. NAC completely reversed the effects of SM, but PQ did not (Fig. 3). This notion was further supported by lipofuscin accumulation increased (Fig. 5), and SOD abolished the effect of SM on Muv phenotype (Supplementary Fig. S9). These results strongly suggested that SM acted as a pro-oxidant to suppress Muv phenotype induced by over-activated *ras*.

In fact, ROS generation increases in cancer cells, further increment of ROS is toxic to cancer cells and finally kills them^49^,^50^. In present work, SM rightly functioned by inducing oxidative stress to inhibit tumor-like symptom induced by over-activated Ras (Figs 3, 4, 5 and 6 and Supplementary Figs S3–9). It was supported by recent works that pro-oxidant kill K-*ras* mutant cancer cell lines^51^,^52^. As for an anti-oxidant SM *in vitro* cause oxidative stress *in vivo*, it may be explained by that SM components could be metabolically converted into compounds no longer anti-oxidant. Of course, it still needs to be investigated in future work.

**Figure 17:**
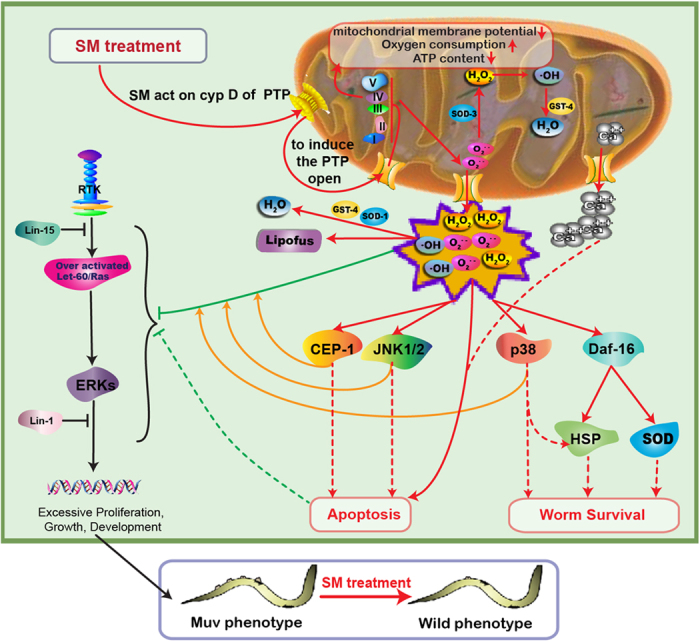
Schematic diagram of the mechanism of SM on suppressing Muv phenotype induced by over-activated Ras. SM regulated CypD of PTP on mitochondrial membrane, induced PTP opening, and triggered oxidative stress to inhibit over-activated Ras/MAPK and promote apoptosis. The p53, JNK, and p38/MAPK were all required for the action of SM. Although SM indeed accelerated anti-oxidant responses, oxidative stress remained predominant throughout and finally killed tumor. Unlike in tumor tissues with high ROS, SM excited oxidative stress to induce predominantly anti-oxidant responses, thus being beneficial to normal organism survival.

The schematic diagram illustrated in Fig. 17 summarized our current view of the action of SM treatment. SM regulated CypD of PTP on mitochondrial membrane, triggered the PTP opening and decreased MMP, which in turn increased oxygen consumption, decreased ATP content, and caused a significant accumulation of ROS. The resultant oxidative stress inhibited over-activated Ras/MAPK and induced gonad cell apoptosis. The p53, JNK, and p38/MAPK pathways were all required for the action of SM in the inhibition of the over-activated Ras/MAPK pathways.

It is obvious that SM induced severe oxidative stress under over-activated *ras* background to alleviate tumor-like symptom in *C. elegans*. It is known that pro-oxidant can trigger mild oxidative stress, and it is healthy beneficial to organisms^53^. In our present work, SM promoted DAF-16 nuclear translocation, and up-regulated its downstream gene expressions of HSP16.2, GST and SODs under wild type ras background (Supplementary Figs S3–9). These results gave evidences on that SM accelerated anti-oxidant responses of worms on a certain degree. Previous work in our lab has demonstrated that SM can extend *C. elegans* lifespan^3^. It is reasonable to believe that SM can specifically kill cancer cells, but not normal cells. Considering our result that SM only inhibited the Muv phenotype of parent worms rather than their progeny, it showed that the effect of SM was at a phenotype level, but not genetic level. Taken together, SM is a promising drug candidate to combat over-activated Ras related cancer.

In conclusion, SM acted as a pro-oxidant to suppress tumor-like symptom induced by over-activated *ras*. It regulated CypD of PTP on mitochondrial membrane to induce the generation superoxide anion, and increase the accumulation of ROS, finally cause severe oxidative stress to suppress the over-activated Ras/MAPK pathway, and p53, JNK, and p38/MAPK were all involved in the action of SM.

## Materials and Methods

### Antibodies and reagents

MAPK Family Antibody Sampler Kit (#9926) and Phospho-MAPK Family Antibody Sampler Kit (#9910) were purchased from Cell Signaling Technology (Beverly, MA). Anti-CEP-1 (cC-18) (sc-135460) antibody was obtained from Santa Cruz Biotechnology (Santa Cruz, CA). Paraquat (PQ), Cyclosporin A (CsA), Bongkrekic acid (BA) and 4,4′-Diisothiocyanatostilbene-2,2′-disulfonic acid disodium salt hydrate (DIDS) were from Sigma. N-acety-L-cysteine (NAC) was purchased from TCI (Shanghai, China). Total Antioxidant Capacity Assay Kit with the ABTS Method (S0119), Fluo-3 AM (S1056), Hoechst 33342 (C1022), Mitochondrial Membrane Potential Assay Kit with JC-1 (C2006), Enhanced BCA Protein Assay Kit (P0010S) and Superoxide dismutase (SOD, S0088) were obtained from Beyotime (Shanghai, China). Acridine orange (AO) was from Dingguo Changsheng Biotechnology (Beijing, China). H_2_DCF-DA (D399), MitoSox (M36008), Mitotracker red (M7512), ATP determination kit (A22066) and SYTO 12 (S7574) were purchased from Molecular Probes (Eugene, Oregon, USA). PVDF membranes and ECL plus detection kit were obtained from Millipore (Bedford, MA, USA).

### Preparation of SM formula

SM formula is composed of RG, RO, and FS (3:3:2) according to Chinese Pharmacopoeia (2015)^3^. RG (9 g), RO (9 g), and FS (6 g) were soaked in 130 mL ddH_2_O for 30 min, and then heated to boil and simmered gently with stirring for 30 min. After cooling at room temperature, the extract was filtered through filter paper, then diluted with ddH_2_O to 50 mL. The concentration is defined as the content of the crude drug in solution (w/v). The solutions were diluted to 3.0 mg/mL (SML), 6.0 mg/mL (SMM), 12.0 mg/mL (SMH), 24.0 mg/mL (SMHH), and 48.0 mg/mL (SMHHH), respectively. Previous work in our lab has shown that the contents of total saponins, flavones, lignin in SM were 0.57 ± 0.04, 0.13 ± 0.00, 0.35 ± 0.00 g/100 g, respectively. The content of marker substances of Rf, Rg2, Rh1, Rg3 and Schizandrin were 19.7 ± 0.1, 74.6 ± 0.5, 34.9 ± 0.1, 103.4 ± 0.6, 3.2 ± 0.3 μg/g, respectively^54^.

### Maintenance and culturing conditions of *C. elegans* strains

CB1275, [*lin-1*(e1275)IV]; TJ356, zIs356 [*daf-16p*::*daf-16a/b*::GFP + *rol-6*]; CF1553, muIs84 [(pAD76) *sod-3p*::GFP + *rol-6*]; AM263, rmIs175 [*unc-54p*::*Hsa-sod-1* (WT)::YFP]; TJ375, gpIs1[*hsp-16-2*::GFP]; CL2166, dvIs19 [(pAF15)*gst-4p*::GFP::NLS] III were provided by the Caenorhabditis Genetics Center (CGC). MT2124, *let-60* (n1046sd, *gf*)IV and MT8189, *lin-15*B(n765ts)X were kindly provided by Howard Hughes Medical Institute. Theses worm strains were maintained by standard methods^55^.

### Drug treatment

All treatments were carried out in 96-well plates. Under normal circumstances, around 80–100 L1 larvae synchronized worms were cultured to adults in 200 μL of S buffer containing SM [0, 3.0 mg/mL (SML), 6.0 mg/mL (SMM), 12.0 mg/mL (SMH), 24.0 mg/mL (SMHH), 48 mg/mL (SMHHH)] with or without 0.5 mM of PQ or 2.5 mM of NAC or 5 μM of CsA or 8 μM of BA or 500 μM of DIDS were transferred to 96-well plates, and 1 mg freshly grown OP50 were added as a standard food source. In Fluo-3 AM, JC-1, SYTO 12, AO and Hoechst 33342 staining experiments, around 80–100 synchronized *let-60(gf)* L3 mutants were incubated with drugs for 48 hours.

### Quantification of the wild type phenotype of mutants

*Let-60 (gf), lin-15* and *lin-1* mutants were treated as described in Drug Treatment part and cultured to adults after 3–4 days, and the percentage of wild type phenotype worms was scored by using inverted microscopy. The percent of wild type phenotype worms were calculated according to the formula: 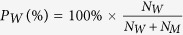; where P_W_ is the percentage of wild type phenotype worms; N_W_ is the number of wild type phenotype worms; N_M_ is the number of Muv phenotype worms^56^,^57^.

### ABTS assay

The experiment was performed to determine the capacity of SM on scavenging ROS *in vitro* according to the protocol of the Total Antioxidant Capacity Assay Kit with the ABTS method^58^.

### SOD activity assay

Treated worms were collected with a M9 buffer and stored at −80 °C. Worms were homogenized with a Pellet Pestle Motor, and the SOD activity assay was evaluated according to the standard NBT^+^ method^59^. The protein in the homogenate was then quantified by the Lowry Method to standardize the SOD activity^60^. The relative specific activity = 
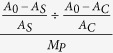
; where A_0_ is the absorbance of blank with light exposure; A_S_ is the absorbance of samples; A_C_ is the absorbance of control; and M_P_ is the protein quantity in the sample divided by the protein quantity in the control.

### Fluorescence quantification and visualization

All fluorescence were visualized under a fluorescence microscope (OLYMPUS, BX53). Mitotracker red was monitored by a confocal microscopy (Leica, SP8). The adult worms were collected with M9 buffer, stained with the relative fluorescence dye. The stained worms were seeded on bacterial lawns for 30 min to reduce the amount of stained bacteria in the gut. Dyes of H_2_DCF-DA, MitoSox, Mitotracker red and Fluo-3 AM were dissolved in DMSO at a high concentration [all at 5 mM except H_2_DCF-DA, which was at 10 mM] and frozen at −20 °C as a stock. MitoSox was prepared freshly for each use. Before staining, stocks were diluted in the M9 buffer by 1:1000^61^. JC-1 kit was used according to the Supplier’s manual. To detect the apoptotic cells in *C. elegans* gonad induced by SM, animals were stained with SYTO 12, AO and Hoechst 33342 as previously described^37^,^38^,^62^. The final concentration of SYTO 12, AO, and Hoechst 33342 were 50 μM, 25 μg/mL and 10 μg/mL, respectively. The adult worms were incubated in SYTO 12 for 4 hours at room temperature. Double staining with AO and Hoechst 33342 was done by incubating animals in AO at 4 °C overnight and washed with M9 buffer for three times, and then incubating in Hoechst 33342 for 4 hours at room temperature. Only animals that stained brightly were scored.

### Oxygen consumption

Young adult worms in 1 mL of M9 buffer were placed into a 3 mL sealed chamber at 21 °C. A fiber-optical oxygen sensor (DW1 Oxygen Electrode Chamber, Hansatech) was inserted into the chamber and the oxygen partial pressure was monitored for 15 to 30 min^61^. After that, worms were collected with M9 buffer then homogenized with a Pellet Pestle Motor. The protein concentration in the homogenate was quantified by the Lowry method to standardize the oxygen consumption.

### ATP content

ATP contents were measured with an ATP determination kit^61^. ATP content value was then normalized by the protein concentration detected by the Lowry method.

### RNA interference

RNAi experiment was carried out in a 96-well plate at 20 °C via feeding the recombinant HT115 (DE3) bacteria as described in the standard protocols^63^. 80–100 synchronized L1 larvae of *let-60(gf)* mutants in a 200 μL of S buffer (containing 100 μg/mL Amp, 5 μg/mL Tet, 1 mM IPTG, 0 or 12 mg/mL SM) were transferred to 96-well plates with 1 mg of freshly grown interference bacteria (induced by 1 mM of IPTG at 37 °C for 4 h) with three replicates. HT115 containing empty L4440 was fed as a control. These animals were cultured to adults for 3–4 days, and the percentage of wild type worms were scored by using inverted microscopy as described above.

### Western blot analysis

Worms were treated as described in drug treatment section and the young adults were collected by M9 buffer after treatment with drugs and/or RNAi. The lysates were obtained by homogenization in RIPA buffer with a Pellet Pestle Motor. Supernatants were collected and total protein concentrations were measured by using an Enhanced BCA Protein Assay Kit. Equal amounts of protein (20 μg) were used for electrophoresis on 12% SDS-PAGE gels and electrophoretically transferred onto PVDF membranes. The membranes were probed overnight at 4 °C with antibody against Erk1/2 (1:2000), phospho-Erk1/2 (1:1000), JNK (1:2000), phospho-JNK (1:1000), p38 (1:2000), phospho-p38 (1:1000), CEP-1 (1:200) and monoclonal anti-GAPDH (1:10000) in TBST containing 1% BSA (w/v). The blots were then incubated for 2 hours with anti-rabbit secondary antibodies. The immune complex was detected by using ECL plus detection kit. The luminescence was visualized on the Tanon-5200 Chemiluminescent Imaging System (Tanon Science and Technology). For the quantification of each protein, their band intensities were normalized by GAPDH as internal reference.

### Statistical and image analysis

The fluorescence signal intensity was quantified using Image J software. The results are presented as the average of three biological replicates. The data was analyzed by one-way ANOVA and Turkey multiple comparison using SPSS 17.0. The bars with the same letters indicated that there was no significant difference among groups. The bars with different letters indicated that there was a significant difference at a level of 0.05 among groups.

## Additional Information

**How to cite this article**: Liu, Y. *et al*. Shengmai Formula suppressed over-activated Ras/MAPK pathway in *C. elegans* by opening mitochondrial permeability transition pore via regulating cyclophilin D. *Sci. Rep.* **6**, 38934; doi: 10.1038/srep38934 (2016).

**Publisher's note:** Springer Nature remains neutral with regard to jurisdictional claims in published maps and institutional affiliations.

## Supplementary Material

Supplementary Information
