# Supplementary material for: Shengmai Formula suppressed over-activated Ras/MAPK pathway in C. elegans by opening mitochondrial permeability transition pore via regulating cyclophilin D
Source: Sci Rep. 2016 Dec 16;6:38934. doi: 10.1038/srep38934 (PMC5159904; doi:10.1038/srep38934)
Supplement: Supplementary Information [file srep38934-s1.pdf]

Title Page

Support Information

Shengmai Formula suppressed over-activated Ras/MAPK pathway in *C. elegans* by opening mitochondrial permeability transition pore via regulating cyclophilin D

Yan Liu[1], Dejuan Zhi[1], Menghui Li[2], Dongling Liu[2], Xin Wang[1], Zhengrong Wu[1], Zhanxin Zhang[1], Dongqing Fei[1],Yang Li[1], Hongmei Zhu[1], Qingjian Xie[2], Hui Yang[3], Hongyu Li[1, 2*]

[1] Gansu high throughput screening and creation center for health products, School of Pharmacy, Lanzhou University, Donggang West Road No.199, Lanzhou 730020, PR China;

[2] Institute of Microbiology, School of Life Sciences, Lanzhou University, Lanzhou 730000, P.R. China;

[3] Institute of Biology, Academy of Sciences, Gansu province.

* Corresponding author:

Prof. Hongyu Li

Lanzhou University, No. 199, Donggang West Road, Lanzhou 730000, P. R. China

Tel: +8613519640428

Fax: 86-931-8915685

E-mail address: lihy@lzu.edu.cn

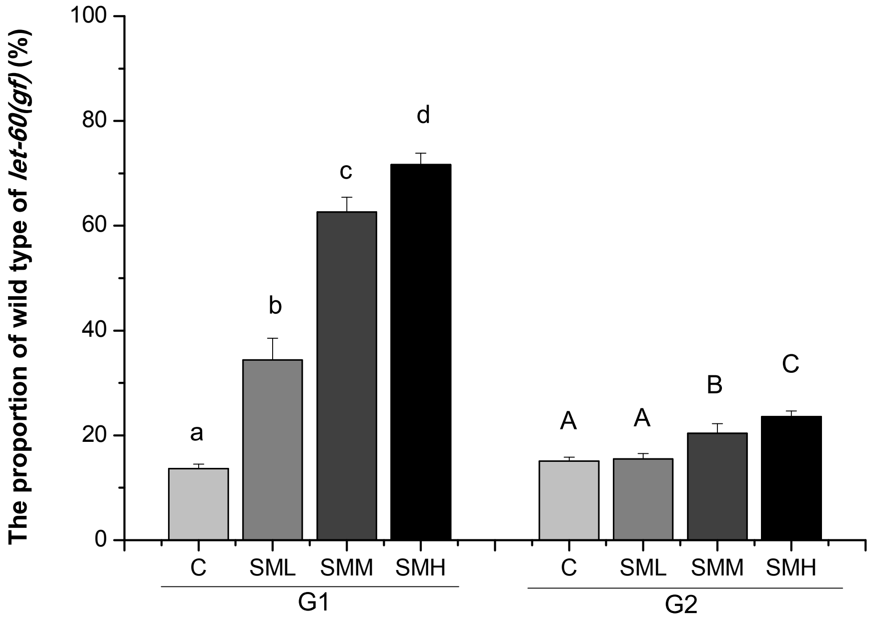

Fig-S. 1

Fig-S. 1 SM inhibited Muv phenotype of *let-60 (gf)* mutant parent worms, and did not alter the tumor-like symptom in their progeny any longer. G1, parent worms treated with SM (N=80-100), G2, the offspring of G1 without SM treatment (N=80-100). C: control. Data is presented as the mean±SD of at least three independent experiments. Bars with different letters indicated that there was a significant difference at a level of 0.05 among groups.

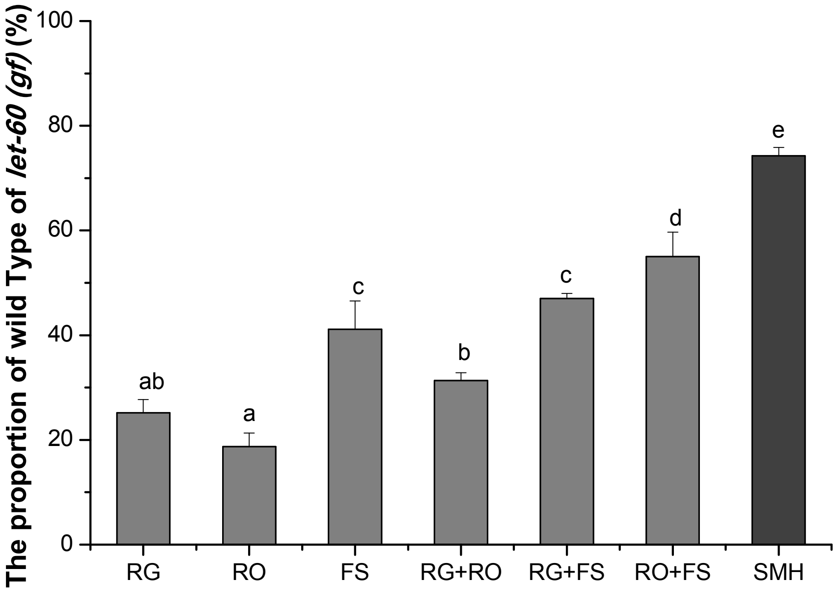

Fig-S. 2

Fig-S. 2 The effect of each herb or all their combinations of SM on the Muv phenotype of *let-60 (gf)* mutants (N=80-100). RG (Radix Ginseng), RO (Radix Ophiopogonis) and FS (Fructus Schisandrae), SMH (RG+RO+FS). The concentration of each herb was equal to that in SMH. Data is presented as the mean±SD of at least three independent experiments. Bars with different letters indicated that there was a significant difference at a level of 0.05 among groups.

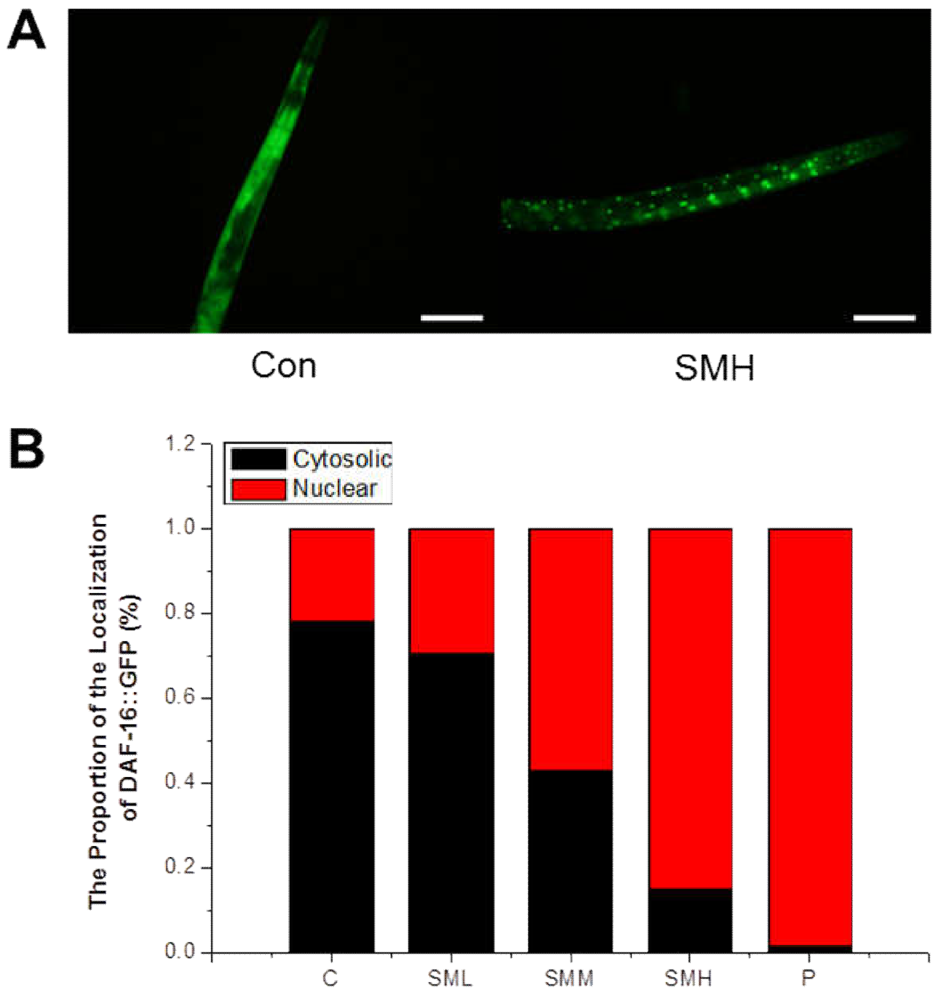

Fig-S. 3

Fig-S. 3 SM promoted DAF-16::GFP nuclear translocation in transgenic strain TJ356. (A) Fluorescence image of worms with or without DAF-16::GFP nuclear translocation (N=20). Con and C: control, P: positive control, heat shocked at 37 °C for 1 hour. Scale bar, 100 μm. (B) Quantified GFP intensity of each group. Data is presented as the mean±SD of at least three independent experiments. Bars with different letters indicated that there was a significant difference at a level of 0.05 among groups.

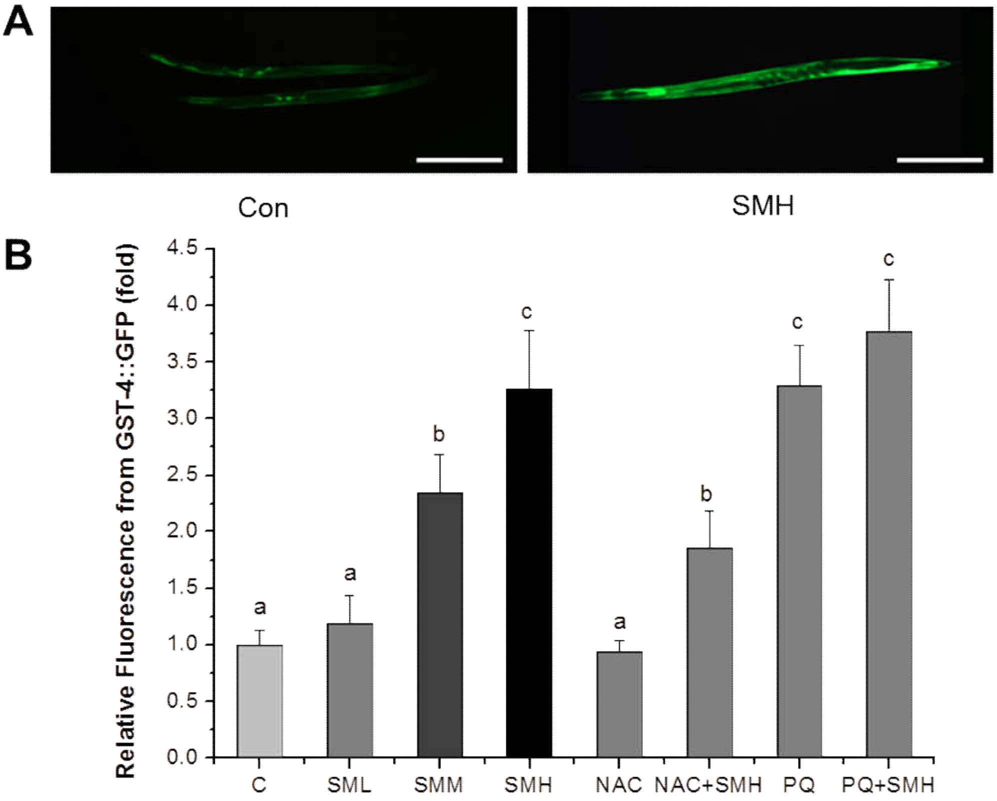

Fig-S. 4

Fig-S. 4 SM increased GST-4::GFP expression in transgenic strain CL2166. (A) Fluorescence image of worms with GST-4::GFP expression (N=20). Con and C: control. NAC, 2.5 mM N-Acety-L-Cysteine; PQ, 0.5 mM paraquat. Scale bar, 200 μm. (B) Quantified GFP intensity of each group. Data is presented as the mean±SD of at least three independent experiments. Bars with different letters indicated that there was a significant difference at a level of 0.05 among groups.

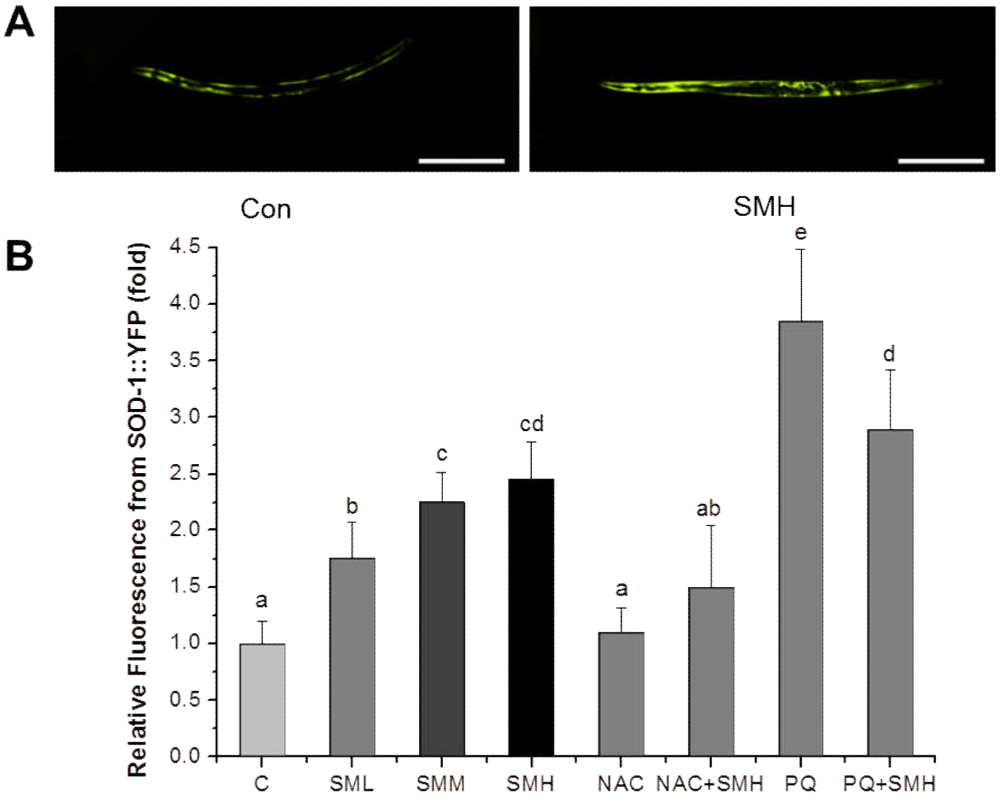

Fig-S. 5

Fig-S. 5 SM enhanced SOD-1::GFP expression in transgenic strain AM263. (A) Fluorescence image of worms with SOD-1::GFP expression (N=20). Con and C: control. NAC, 2.5 mM N-Acety-L-Cysteine; PQ, 0.5 mM paraquat. Scale bar, 200 μm. (B) Quantified GFP intensity of each group. Data is presented as the mean±SD of at least three independent experiments. Bars with different letters indicated that there was a significant difference at a level of 0.05 among groups.

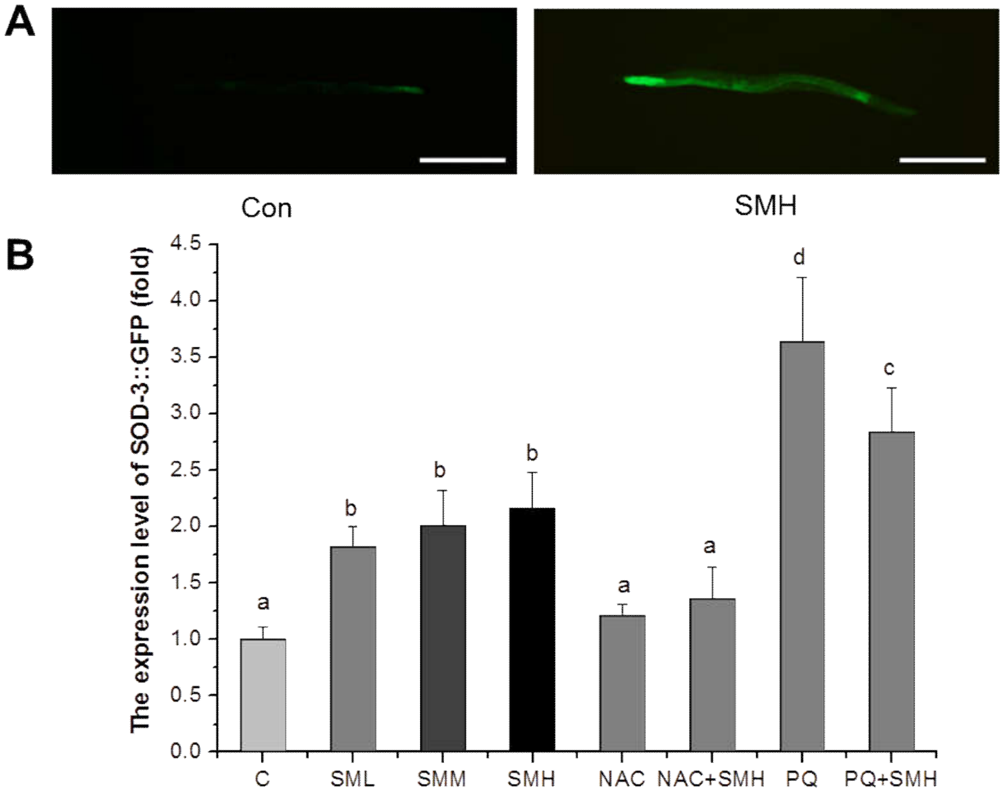

Fig-S. 6

Fig-S. 6 SM up-regulated SOD-3::GFP expression in transgenic strain CF1553. (A) Fluorescence image of worms with SOD-3::GFP expression (N=20). Con and C: control. NAC, 2.5 mM N-Acety-L-Cysteine; PQ, 0.5 mM paraquat. Scale bar, 200 μm. (B) Quantified GFP intensity of each group. Data is presented as the mean±SD of at least three independent experiments. Bars with different letters indicated that there was a significant difference at a level of 0.05 among groups.

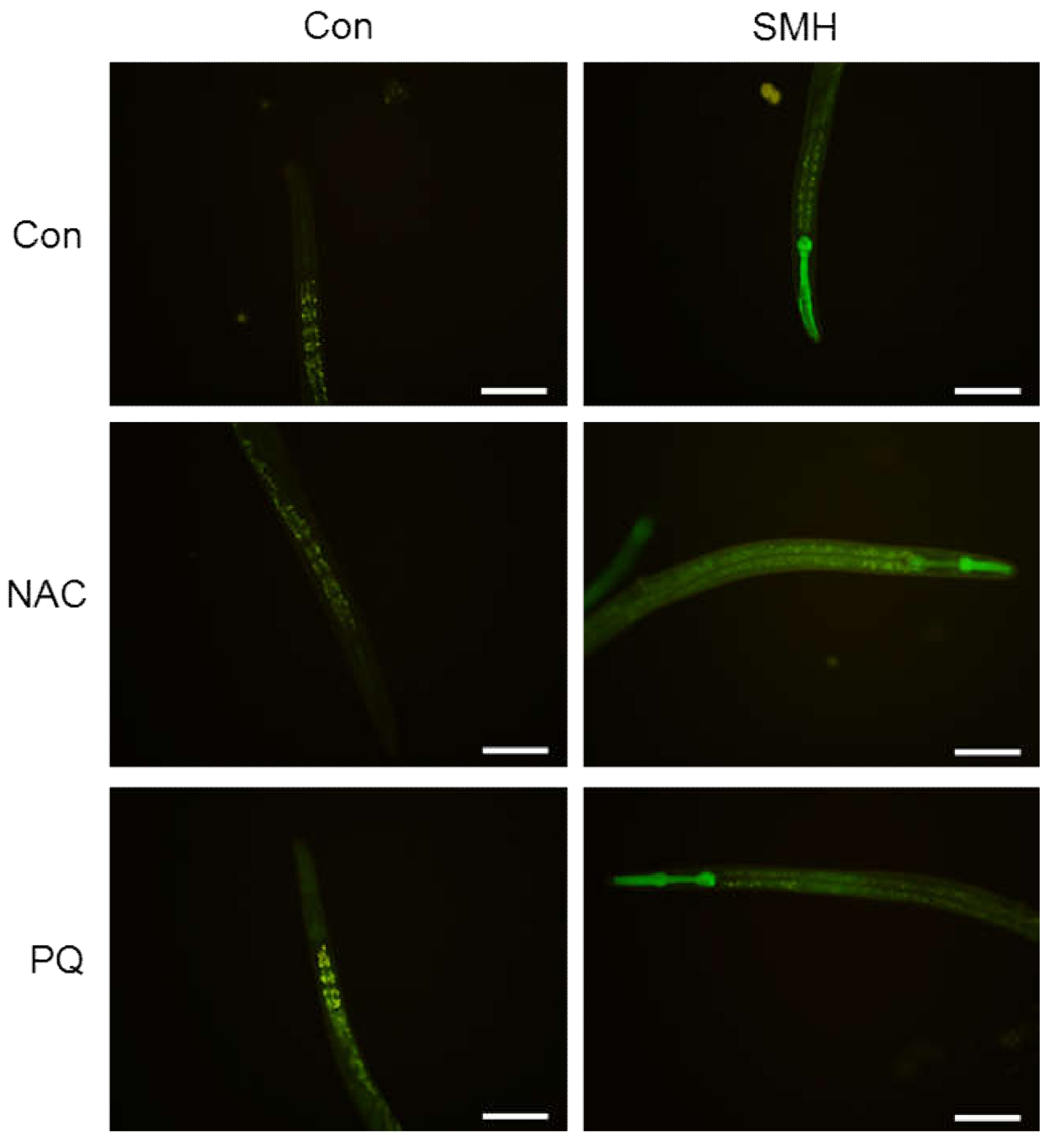

Fig-S. 7

Fig-S. 7 SM induced heat shock protein HSP-16.2::GFP expression in transgenic strain TJ375 (N=20). Con and C: control. NAC, 2.5 mM N-Acety-L-Cysteine; PQ, 0.5 mM paraquat. Scale bar, 100 μm. Data is presented as the mean±SD of at least three independent experiments. Bars with different letters indicated that there was a significant difference at a level of 0.05 among groups.

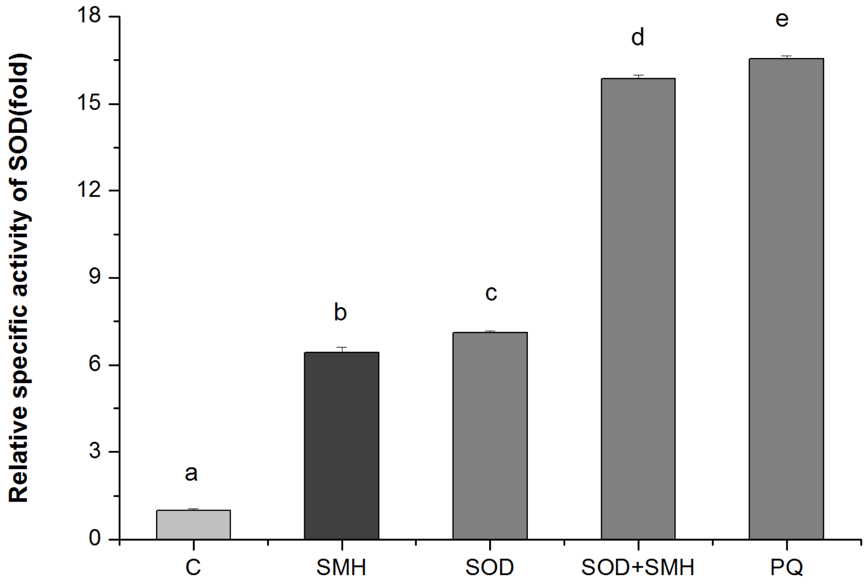

Fig-S. 8

Fig-S. 8 SM increased SOD activity in *let-60(gf)* mutants. C: control. SOD, 150 U superoxide dismutase. PQ, 0.5 mM paraquat. Data is presented as the mean±SD of at least three independent experiments. Bars with different letters indicated that there was a significant difference at a level of 0.05 among groups.

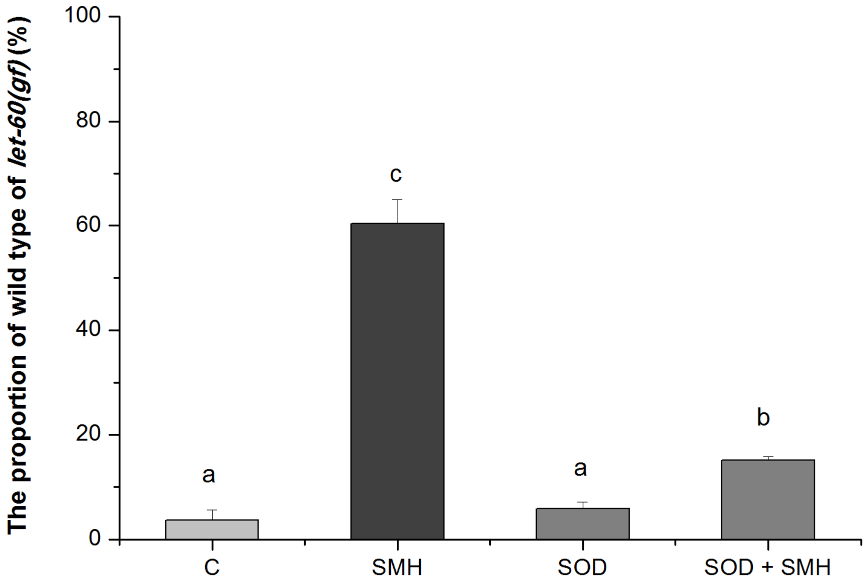

Fig-S. 9

Fig-S. 9 The exogenous SOD reverted the effect of SM on the Muv phenotype of *let-60(gf)* mutants (N=80-100). C: control. SOD, 150 U superoxide dismutase. Data is presented as the mean±SD of at least three independent experiments. Bars with different letters indicated that there was a significant difference at a level of 0.05 among groups.

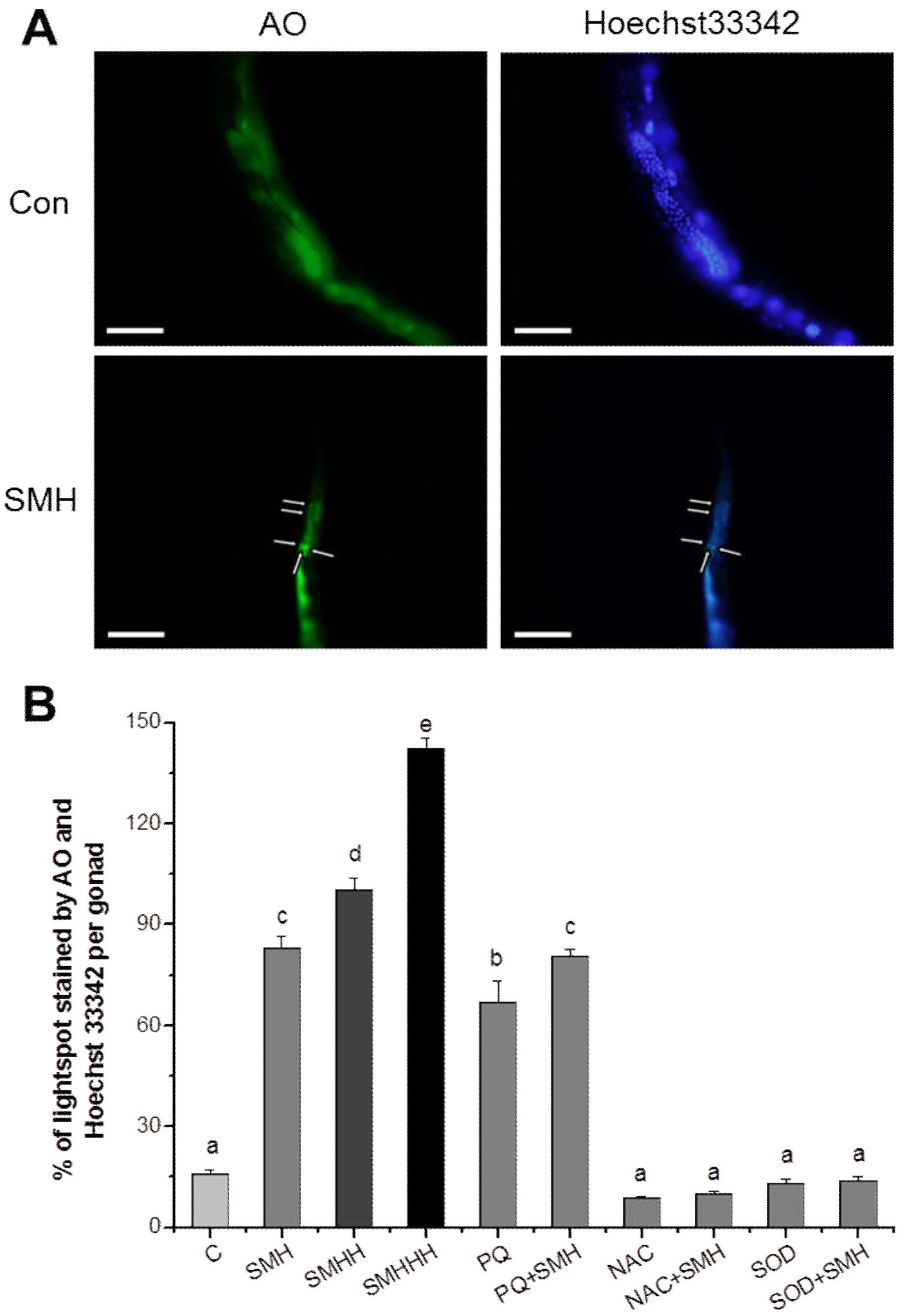

Fig-S. 10

Fig-S. 10 SM enhanced germ cell apoptosis in *let-60 (gf)* mutants. (A) Worms were stained by OA/Hoechst 33342 double staining (25μg/mL and 10 μg/mL, respectively) after treated with SM for 48 hours. Germ cell corpse was indicated by white arrows. Con and C: control. SMH, 12 mg/mL; SMHH, 24 mg/mL; SMHHH, 48 mg/mL. NAC, 2.5 mM N-Acety-L-Cysteine; PQ, 0.5 mM paraquat. Scale bar, 50 μm. (B) Quantified the number of germ cell corpse of each group. Data is presented as the mean±SD of at least three independent experiments. Bars with different letters indicated that there was a significant difference at a level of 0.05 among groups.

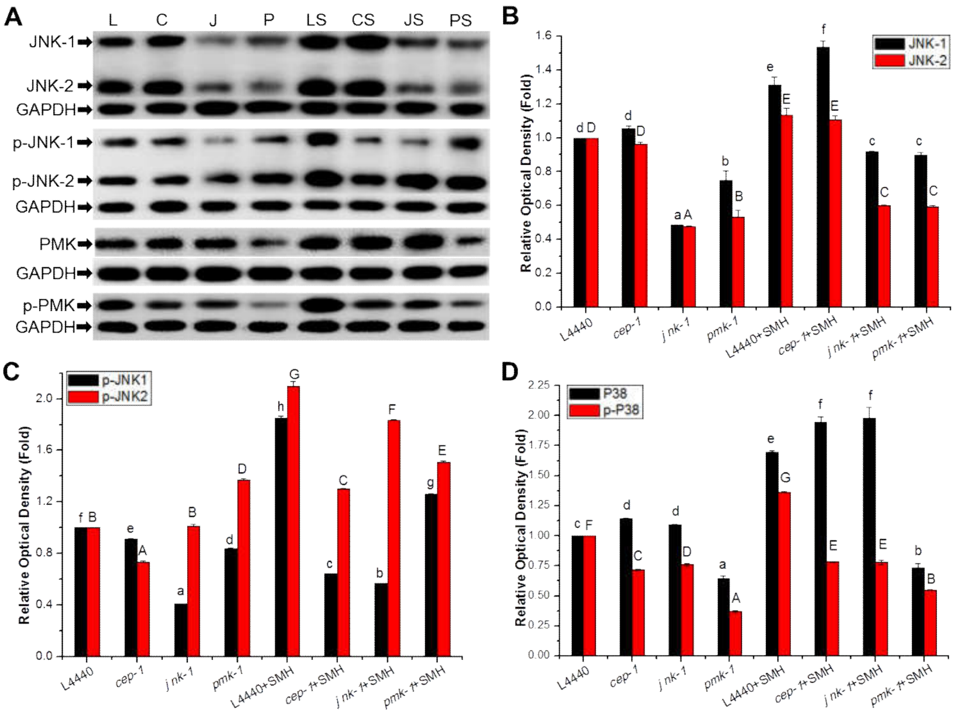

Fig-S. 11

Fig-S. 11 JNK and p38 MAPK signal pathway was involved in SM suppressing over-activated Ras/MAPK pathway. (A) The effect of SM on JNK1/2, p- JNK1/2, P38 and p-P38 in *let-60 (gf)* mutants. Quantification of JNK-1/2 (B), p-JNK1/2 (C), P38 and p-P381/2 (D) band intensities, which were normalized using GAPDH blots. L, C, J, P, LS, CS, JS and PS were groups treated by RNAi of L4440, *cep-1*, *jnk-1*, *pmk-1*, L4440+SMH, *cep-1*+SMH, *jnk-1*+SMH and *pmk-1*+SMH, respectively. Data is presented as the mean±SD of at least three independent experiments. Bars with different letters indicated that there was a significant difference at a level of 0.05 among groups.

Group	Concentration (mg/mL)	*lin-15 (lf)* mutants		*lin-1 (lf)* mutants	
		Muv(%)	N	Muv(%)	N
Control	0	100	77	100	86
SM	3.0	100	72	100	85
	6.0	100	69	100	83
	12.0	100	78	100	87

Tab-S. 1

Tab-S. 1 SM has no effect on the Muv phenotype of *lin-15* and *lin-1* mutants.
